# *ADAMTS9-AS2* acts as an epigenetic brake to constrain DNMT3B-mediated *CADM2* silencing in esophageal squamous cell carcinoma metastasis

**DOI:** 10.3389/fimmu.2026.1752827

**Published:** 2026-03-06

**Authors:** Fang-Fang Shen, Dong-Fen Li, Ling-Bei Kong, Jia-Le Li, Shi-Long Ma, Hao-Zhe Jiang, Hao-Ze Yuan, Yan Jin, Zhi-Guo Chen, Xiu-Juan Guo, Gao-Pan Dong, De-Rong Lu, Jia-Teng Zhong

**Affiliations:** 1 The Key Laboratory for Tumor Translational Medicine, The Third Affiliated Hospital of Xinxiang Medical University, Xinxiang, Henan, China; 2 School of Basic Medical Sciences, Xinxiang Medical University, Xinxiang, Henan, China; 3 School of Pharmacy, Xinxiang Medical University, Xinxiang, Henan, China; 4 Department of Gastroenterology, The Third Affiliated Hospital of Xinxiang Medical University, Xinxiang, Henan, China; 5 Department of Pathology, Xinxiang Medical University, Xinxiang, Henan, China

**Keywords:** ADAMTS9-AS2, CADM2, DNMT3B, epigenetic therapy, esophageal squamous cell carcinoma, metastasis

## Abstract

**Introduction:**

Metastatic recurrence drives dismal survival in esophageal squamous cell carcinoma (ESCC), yet epigenetic mechanisms underlying metastasis remain poorly defined. While DNMT1 and DNMT3A contribute to ESCC pathogenesis, DNMT3B’s role is enigmatic despite frequent dysregulation.

**Methods:**

Integrated methylome-transcriptome profiling comprised genome-wide methylation screening in 5 paired ESCC tumor and adjacent normal tissues. Parallel mRNA microarray profiling quantified expression levels of *DNMT3B*, *CADM2*, and *ADAMTS9-AS2* in ESCC tumors. RIP, ChIP, and pyrosequencing in ESCC cells validated molecular interactions.

**Results:**

*ADAMTS9-AS2* downregulation promoted ESCC proliferation, migration, and invasion. Mechanistically, *ADAMTS9-AS2* directly bound DNMT3B, preventing its occupancy at the *CADM2*. Rescue experiments confirmed CADM2 overexpression reversed *ADAMTS9-AS2* knockdown-induced oncogenic phenotypes. Clinically, DNMT3B overexpression in lymph node-positive tumors correlated with metastatic progression.

**Discussion:**

*ADAMTS9-AS2* functions as an epigenetic brake by sequestering DNMT3B, thereby blocking *CADM2* epigenetic silencing and metastasis in ESCC. Targeting this axis offers potential therapeutic strategies against ESCC.

## Graphical Abstract

**Figure:**
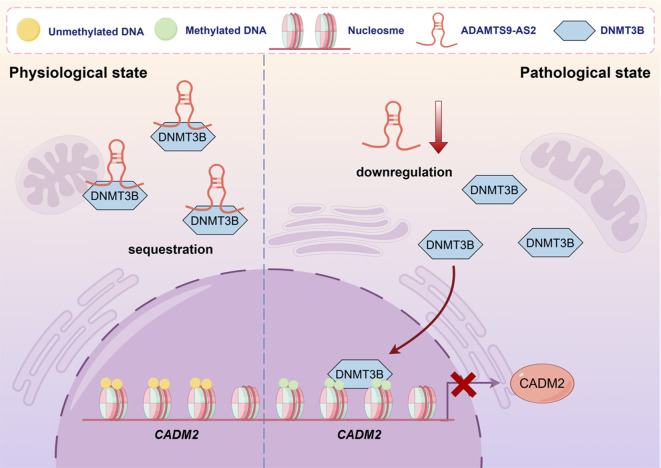
In the physiological condition (left panel), the lncRNA *ADAMTS9-AS2* sequesters DNMT3B, preventing its binding to the *CADM2* gene and thereby maintaining a transcriptionally permissive, hypomethylated state. In the ESCC pathological condition (right panel), the downregulation of *ADAMTS9-AS2* releases DNMT3B, which subsequently binds to *CADM2*, leading to CpG hypermethylation and epigenetic silencing of the gene. This DNMT3B-mediated silencing of *CADM2* ultimately​ drives ESCC metastasis.

## Highlights

- *ADAMTS9-AS2* sequesters DNMT3B to block *CADM2* silencing.
- DNMT3B escalates in lymph node-positive ESCC tumors.
- Targeting *ADAMTS9-AS2*/DNMT3B axis suppresses ESCC metastasis.

## Introduction

Esophageal squamous cell carcinoma (ESCC) represents a global health burden with particularly high incidence in Eastern Asia (1). Advanced ESCC patients face dismal survival rates primarily due to metastatic recurrence, driven partly by epigenetic dysregulation (2). DNA methyltransferases (DNMTs), including DNMT1, DNMT3A, and DNMT3B, catalyze DNA methylation, a key epigenetic mechanism for gene expression regulation (3). While DNMT1 and DNMT3A roles in ESCC are partially characterized (4, 5), DNMT3B’s function remains unexplored despite its frequent dysregulation.

CADM2 is a well-established tumor suppressor in prostate (6) and renal cell carcinomas (7). In ESCC, Shen et al. demonstrated concurrent downregulation of *ADAMTS9-AS2* and CADM2, both correlating with poor prognosis and serving as independent prognostic markers (8). Existing studies reveal CADM2 suppression via miRNA-mediated mechanisms in ESCC. For example, Li et al. reported that miR-21-5p directly targets CADM2 to promote ESCC proliferation (9). Zhu et al. demonstrated that miR-182-5p suppresses CADM2 to accelerate invasion via Akt signaling in ESCC (10). Critically, our data showed that DNMT3B expression inversely correlated with both *ADAMTS9-AS2* and CADM2, suggesting a novel regulatory axis beyond miRNA pathways.

We hypothesize that *ADAMTS9-AS2* functions as an epigenetic brake by sequestering DNMT3B, thereby influencing *CADM2* methylation, which in turn modulates its tumor-suppressive activity and ultimately constrains ESCC progression. Supporting this, *ADAMTS9-AS2* is recognized as a tumor-suppressive lncRNA that modulates DNMT activity in cancers (11), though its interaction with DNMT3B remains uncharacterized.

To define the functional impact of *ADAMTS9-AS2* guided by our integrated methylome-transcriptome data, we employed loss-of-function experiments in ESCC metastasis. We further elucidated how it regulates *CADM2* methylation via its interaction with DNMT3B using RIP, ChIP, and pyrosequencing assays. Clinical validation across primary tumors and lymph node metastases establishes the translational relevance of this lncRNA-guided epigenetic axis.

## Materials and methods

### Cell culture

All human ESCC cell lines, including 9706, KYSE150, EC-1, KYSE30, KYSE70, and TE-1, and the immortalized normal human esophageal epithelial cell line Het-1A were obtained from Department of Gastroenterology and Hepatology, Chinese PLA General Hospital. All cell lines were confirmed to be free of mycoplasma contamination by PCR-based testing. Where applicable (excluding syngeneic lines), cell lines were authenticated by short tandem repeat (STR) profiling. ESCC cells were cultured in RPMI-1640 medium supplemented with 10% fetal bovine serum (FBS) and 1% penicillin-streptomycin. Het-1A cells were maintained in bronchial epithelial cell growth medium (BEGM) supplemented with the BulletKit™ growth factors and 1% penicillin-streptomycin. All cell lines were incubated at 37 °C in a humidified atmosphere containing 5% CO_2_.

### siRNA and plasmid transfection

Gene knockdown and overexpression experiments were performed using Lipofectamine 3000 transfection reagent (Invitrogen) according to the manufacturer’s instructions. For siRNA-mediated knockdown, si-*ADAMTS9-AS2*, si-*DNMT3B*, si-*DNMT1* and negative control si-NC were all synthesized by GenePharma (Shanghai, China). To enhance knockdown efficiency and minimize off-target effects, a pool of three distinct siRNAs targeting different sequences of the same gene was used for transfection. For plasmid overexpression, pcDNA3.1-*CADM2*, pcDNA3.1-*ADAMTS9-AS2*, pcDNA3.1-*DNMT3B*, pcDNA3.1-*DNMT1*, or empty vector pcDNA3.1 were constructed. Transfected cells were incubated for 48 h at 37 °C before functional assays. The siRNA sequences for si-*ADAMTS9-AS2*, si-*DNMT3B*, and si-*DNMT1* are shown in **Table 1**.

**Table 1:** The primer sequences for siRNA.

siRNA	sequence (5’-3’)
si-*ADAMTS9-AS2*#1	GUGCUGUCCUUUUGUAGUCUTT
si-*ADAMTS9-AS2*#2	CGGCUUUCAAGAUUGGAAUTT
si-*ADAMTS9-AS2*#3	CAGAGACGCAGGUAUUUAUTT
si-*DNMT3B*#1	GCCCAUUUGACUUGGUGAUTT
si-*DNMT3B*#2	GGAGCUGUUACAUGUGUCUTT
si-*DNMT3B*#3	GUACCAUGCUCUGGAGAAATT
si-*DNMT1*#1	GGAAGAAGAGUUACUAUAATT
si-*DNMT1*#2	GCUUCAGUGUGUACUGUAATT
si-*DNMT1*#3	GAAGAAGCACAGAAGUCAATT

### Reverse transcription quantitative PCR

Total RNA was isolated from cultured cells using the RNAeasy™ Animal RNA Extraction Kit (Beyotime) according to the manufacturer’s protocol. RNA concentration and purity were determined spectrophotometrically (NanoDrop 2000, Thermo Scientific) with A260/A280 ratios maintained between 1.8-2.0. Genomic DNA contamination was eliminated by DNase I treatment (Thermo Scientific). First-strand cDNA synthesis was performed using 1 μg total RNA with the SweScript RT II First Strand cDNA Synthesis Kit (Servicebio) in 20 μL reactions containing oligo(dT) primers and RT enzyme under thermal conditions of 25 °C for 5 min, 55 °C for 15 min, and 85 °C for 5 s. Quantitative PCR amplification was carried out using SYBR^®^ Select Master Mix (Applied Biosystems) on a QuantStudio 6 Flex Real-Time PCR System (Applied Biosystems). Reactions contained 10 μL master mix, 1 μL cDNA template, 0.8 μL gene-specific primers (10 μM), and nuclease-free water to a final volume of 20 μL. The thermal cycling protocol consisted of an initial denaturation at 95 °C for 10 min, followed by 40 cycles of 95 °C for 10 s and 60 °C for 60 s. Gene expression levels were normalized to the endogenous reference gene *GAPDH* to allow direct comparability between lncRNA and mRNA expression within the same samples, and calculated using the 2^-ΔΔCt^ method. All RT-qPCR experiments were performed with three independent biological replicates, each with triplicate technical repeats. Primer sequences were synthesized by Tsingke Biotechnology (Beijing, China) and listed as follows.

*ADAMTS9-AS2*-Forward primer (F): TTTACCATGCGCTGAGTGAG.
*ADAMTS9-AS2*-Reverse primer (R): AAAGTTGCGTCATGCTTCGG.
*CADM2*-F: CCTCAATGCCACCCCTCAG.
*CADM2*-R: TTCTCCGCCATCCTTTGTCC.
*DNMT3B*-F: GCAAAGACCGAGGGGATGAA.
*DNMT3B*-R: CCTGCCACAAGACAAACAGC.
*GAPDH*-F: CATGAGAAGTATGACAACAGCCT.
*GAPDH*-R: AGTCCTTCCACGATACCAAAGT.

### Cell counting kit-8 assay

Cell proliferation was quantified using the Enhanced CCK-8 Kit (Beyotime) following standardized protocols. ESCC cells were seeded into 96-well plates at a density of 5×10^3^ cells per well in 100 μL complete medium and incubated overnight at 37 °C under 5% CO_2_ to allow attachment. After transfection, cells were cultured for 0, 24, 48, 72, or 96 h. At each time point, 10 μL of CCK-8 reagent was added directly to each well followed by incubation for 2 h at 37 °C protected from light. The absorbance at 450 nm was measured using a SpectraMax M5 microplate reader (Molecular Devices) with a reference wavelength of 650 nm to correct for optical imperfections.

### Cell cycle analysis by flow cytometry

Cell cycle distribution was assessed using propidium iodide (PI) DNA staining. Briefly, ESCC cells were harvested 48 h post-transfection, washed twice with ice-cold PBS, and fixed in 70% ethanol at 4 °C overnight. Fixed cells were pelleted by centrifugation at 300 ×g for 5 min, resuspended in 500 μL PBS containing 50 μg/mL PI (Sigma-Aldrich) and 100 μg/mL RNase A (Thermo Scientific), and incubated at 37 °C for 30 min protected from light. Samples were filtered through 40-μm nylon mesh to remove aggregates prior to analysis. Flow cytometry was performed on a BD FACSCanto II system (BD Biosciences) equipped with a 488-nm argon laser. PI fluorescence was collected using a 585/42 nm bandpass filter. The percentage of cells in G1, S, and G2/M phases were quantified based on DNA content. Three independent biological replicates were analyzed per experimental condition.

### Apoptosis detection by flow cytometry

Apoptotic rates were quantified using dual staining with Annexin V-APC and 7-aminoactinomycin D (7-AAD) according to standardized protocols. ESCC cells were harvested 48 h post-transfection, washed twice with ice-cold PBS, and resuspended in 100 μL 1× binding buffer (BD Biosciences). Cells were stained with 5 μL Annexin V-APC and 5 μL 7-AAD solution for 15 min at room temperature protected from light. Subsequently, 400 μL binding buffer was added to each sample prior to immediate analysis. Flow cytometry was performed on a BD FACSCanto II system (BD Biosciences) equipped with 488-nm and 633-nm lasers. Annexin V-APC fluorescence was detected using a 660/20 nm bandpass filter, while 7-AAD emission was collected through a 585/42 nm filter. Viable cells were defined as Annexin V^-^/7-AAD^-^, early apoptotic as Annexin V^+^/7-AAD^-^, late apoptotic as Annexin V^+^/7-AAD^+^, and necrotic as Annexin V^-^/7-AAD^+^. Data analysis utilized FlowJo software (BD Life Sciences) with quadrant gating. Three independent biological replicates were performed per condition.

### Wound healing assay

Cell migration capacity was evaluated using an *in vitro* scratch wound healing model. ESCC cells were seeded into 6-well plates at a density of 5×10^5^ cells per well and cultured in complete medium until reaching 90-100% confluency. A sterile 200-μL pipette tip was used to create wounds by vertically scratching the cell monolayer. Detached cells and debris were removed by washing twice with PBS. Cells were then incubated in serum-free medium to minimize proliferation interference. Wound closure was monitored at 0, 24, and 48 h post-scratching. The wound area at different time points was quantified by ImageJ software (NIH).

### Transwell migration and invasion assay

Cell migration and invasive capacities were assessed using Corning Transwell chambers with 8-μm pore polycarbonate membranes (Corning). For Transwell migration assay, membranes were used without coating. For Transwell invasion assay, Matrigel matrix (BD Biosciences) was diluted and applied to the upper chamber (50 μL/insert), followed by polymerization at 37 °C for 2 h. ESCC cells were serum-starved for 12 h, harvested, and resuspended in serum-free medium at 5×10^4^ cells/mL. Cell suspensions (200 μL) were seeded into the upper chamber, while 600 μL complete medium containing 20% FBS was added to the lower well as a chemoattractant. After 24 h incubation at 37 °C with 5% CO_2_, non-migrating/invading cells on the upper membrane surface were removed with cotton swabs. Migrated or invaded cells on the lower surface were fixed with 4% paraformaldehyde for 20 min and stained with 0.1% crystal violet (Beyotime) for 15 min. Three random fields per membrane were imaged using an Olympus BX53 microscope. Migrated or invaded cells were quantified using ImageJ software with the Cell Counter plugin.

### Western blot analysis

Protein expression was evaluated by Western blotting following standardized protocols. Cultured cells were lysed in RIPA buffer (Beyotime) supplemented with 1% protease inhibitor cocktail (Roche) on ice for 30 min. Lysates were centrifuged at 12,000 ×g for 15 min at 4 °C, and supernatants were collected. Protein concentrations were determined using the BCA Protein Assay Kit (Thermo Scientific) with bovine serum albumin as standard. Equal amounts of protein (30 μg per lane) were separated by 12% SDS-PAGE and transferred onto PVDF membranes (Millipore). Membranes were blocked with 5% non-fat milk in TBST for 1 h at room temperature and incubated overnight at 4 °C with primary antibodies diluted in blocking buffer. Antibodies included anti-CADM2 (Solarbio, Beijing, #K009282P), anti-E-cadherin (Proteintech, Wuhan, #20874-1-AP), anti-N-cadherin (Proteintech, Wuhan, #22018-1-AP), anti-Vimentin (Proteintech, Wuhan, #10366-1-AP), and anti-β-actin (Proteintech, Wuhan, #20536-1-AP). After three TBST washes, membranes were incubated with HRP-conjugated secondary antibodies for 1 h at room temperature. Protein bands were visualized using enhanced chemiluminescence substrate (Millipore) and imaged on a ChemiDoc MP Imaging System (Bio-Rad). Band intensities were quantified using ImageJ Software with β-actin as loading control.

### Clinical specimen collection

All human tissue specimens were obtained with written informed consent from patients undergoing surgical resection for ESCC at the Third Affiliated Hospital of Xinxiang Medical University, under protocols approved by the Institutional Review Board of the Third Affiliated Hospital of Xinxiang Medical University (K2024117-01). The inclusion criteria for this study were (1): histologically confirmed primary ESCC (2); patients undergoing curative-intent surgical resection without prior neoadjuvant therapy (3); availability of complete clinical-pathological data. Exclusion criteria included: (1) history of other malignancies; (2) receipt of preoperative chemotherapy or radiotherapy. Specimens were collected from two independent cohorts. The sample size for Cohort 1 (n=24 pairs) was determined based on the standard cohort size used in discovery-phase epigenetic studies in ESCC, providing >80% power to detect methylation differences with an effect size of >0.8 at α=0.05. Cohort 2 (n=10 triplet sets of specimens) was designed as a validation set.

Specimens were collected from two independent cohorts. Cohort 1 comprised 24 paired fresh tissues including histologically confirmed ESCC tumors and matched adjacent normal mucosa (collected ≥5 cm from the tumor margin), which were immediately snap-frozen in liquid nitrogen within 15 min of resection and stored at -80 °C for Agena methylation. All adjacent normal tissues underwent rigorous histological verification by two independent pathologists to confirm the absence of tumor cells and dysplastic changes; only tissues with confirmed normal squamous epithelium and underlying submucosal architecture were included. Cohort 2 included 10 triplet sets of specimens, with each set comprising the primary tumor, adjacent normal tissue, and a lymph node metastasis from the same patient. The same histological verification process was applied to all Cohort 2 specimens. The detailed clinicopathological characteristics of Cohort 1 and Cohort 2 are summarized in **Supplementary Tables 1**, **2**, respectively.

### Genome-wide methylation analysis

Genome-wide DNA methylation profiling was performed using Illumina methylation microarrays (850K CpG sites). Genomic DNA extracted from 5 paired ESCC tumor and adjacent normal fresh-frozen tissues underwent bisulfite conversion using the EZ DNA Methylation-Lightning Kit (Zymo Research). Converted DNA was amplified, fragmented, and hybridized to the BeadChip according to the manufacturer’s protocol. Arrays were scanned on an Illumina iScan system, and raw intensity data were processed using the ChAMP package in R (v4.0.3) for quality control, normalization (BMIQ), which inherently adjusts for technical variations and potential batch effects, and β-value calculation. Differentially methylated positions (DMPs) were identified with a threshold of |Δβ|≥0.2 and adjusted *P* < 0.05, with *P*-values adjusted for multiple testing using the Benjamini-Hochberg false discovery rate (FDR) method as a standard procedure.

### Targeted methylation validation by MassARRAY EpiTYPER

Targeted DNA methylation validation was performed using the MassARRAY EpiTYPER platform (Agena Bioscience) on 24 paired ESCC tumor and adjacent normal tissues. Genomic DNA underwent bisulfite conversion (EZ DNA Methylation-Lightning Kit, Zymo Research), followed by PCR amplification of the *CADM2* locus flanking the cg03455765 region. Amplified products were processed through *in vitro* transcription base-specific cleavage and analyzed by matrix-assisted laser desorption/ionization time-of-flight mass spectrometry. Methylation levels at 17 individual CpG sites within the cg03455765 amplicon were quantified as β-values using EpiTYPER software. cg03455765 sequences were F: 5’- GTTTGAATGTTGTTGATGGAATTTT-3’, R: 5’- AAAACAATCTCACAAAAACCCTACTT-3’. Statistical significance was assessed by paired t-test (*P* < 0.05).

### Pyrosequencing analysis

Pyrosequencing was employed to assess the functional impact of *ADAMTS9-AS2* on the DNA methylation level of the *CADM2* gene CpG island. Genomic DNA from TE-1 cells transfected with si-*ADAMTS9-AS2* or EC-1 cells transfected with pcDNA3.1-*ADAMTS9-AS2* underwent bisulfite conversion using the EZ DNA Methylation-Lightning Kit (Zymo Research). Biotinylated PCR amplicons spanning the target region of the *CADM2* were sequenced on a PyroMark Q48 Autoprep system (Qiagen).

### Whole transcriptome microarray analysis

Whole transcriptome profiling was performed using Agilent SurePrint G3 Human GE v3 microarrays (8×60K) on 5 paired ESCC tumor and adjacent normal tissues. Total RNA was extracted with TRIzol reagent (Invitrogen), quantified (NanoDrop), and quality-controlled. RNA amplification, labeling with Cy3/Cy5 dyes (Quick Amp Labeling Kit), and hybridization followed manufacturer protocols. Arrays were scanned on an Agilent G2565CA scanner. Raw data were processed with Feature Extraction Software, normalized by quantile method, and subsequently adjusted for potential batch effects using the ComBat function from the sva package in R. Processed data were analyzed using the limma package. Differentially expressed genes (DEGs) were identified with |log_2_FC|≥2 and adjusted *P* < 0.05, employing the Benjamini-Hochberg FDR method for multiple testing correction. Heatmap visualization displayed expression patterns of target genes (DNMT1, DNMT3A, DNMT3B, CADM2, ADAMTS9-AS2).

### RNA immunoprecipitation assay

RNA-protein interactions were analyzed using the Magna RIP Kit (Millipore). EC-1 cells were lysed in RIP buffer containing protease/RNase inhibitors. Lysates were incubated overnight at 4 °C with 5 μg anti-DNMT3B antibody (Proteintech, Wuhan, #26971-1-AP) or IgG control conjugated to magnetic beads. After washes, bound RNAs were extracted using TRIzol and reverse-transcribed into cDNA, and *ADAMTS9-AS2* enrichment was quantified by RT-qPCR using the specific primers: Forward primer (5’-ATGTCTGGCTGAAAGCCGAA-3’) and Reverse primer (5’-GGAGCAGGTCCAGGTGTTAC-3’). Data were normalized to input controls and expressed as fold-change relative to IgG.

### RNA pull-down assay

To validate the direct interaction between *ADAMTS9-AS2* and DNMT3B protein, an RNA pull-down assay was performed. Briefly, biotin-labeled sense and antisense (control) RNA probes corresponding to the full-length *ADAMTS9-AS2* transcript were synthesized *in vitro* using a biotin RNA labeling kit and confirmed by streptavidin-HRP blot. Whole-cell lysates were prepared from TE-1 cells using RIPA buffer supplemented with protease and RNase inhibitors. The lysates were incubated with streptavidin-coated magnetic beads pre-bound with the biotinylated RNA probes. After extensive washing to remove non-specifically bound proteins, the RNA-protein complexes were eluted and subjected to SDS-PAGE followed by western blot analysis using an anti-DNMT3B antibody to detect specifically pulled-down proteins.

### Subcellular fractionation and RNA localization

To determine the subcellular localization of *ADAMTS9-AS2*, nuclear and cytoplasmic RNA fractions were isolated from TE-1 cells using a commercially available PARIS Kit, following the manufacturer’s protocol. Total RNA was extracted from each fraction using TRIzol reagent. RNA concentration and purity were assessed with a NanoDrop spectrophotometer. First-strand cDNA was synthesized from equal amounts of nuclear and cytoplasmic RNA using a reverse transcription kit with gDNA removal. The relative enrichment of *ADAMTS9-AS2* in nuclear versus cytoplasmic fractions was then quantified by real-time qPCR using gene-specific primers. *U6* small nuclear RNA and *GAPDH* mRNA were used as nuclear and cytoplasmic reference controls, respectively. The 2^-ΔΔCt^ method was applied to calculate the relative abundance of *ADAMTS9-AS2* in each compartment.

### Chromatin immunoprecipitation

ChIP was performed using the Magna ChIP Kit (Millipore). EC-1 cells transfected with si-*ADAMTS9-AS2* or si-NC were cross-linked with 1% formaldehyde for 10 min at room temperature. Chromatin was sheared to 200–500 bp fragments by sonication and immunoprecipitated overnight at 4 °C with 5 μg anti-DNMT3B antibody (Proteintech, Wuhan, #26971-1-AP) or IgG control. Precipitated DNA was purified and quantified by qPCR using SYBR Green Master Mix (Applied Biosystems). The primers of *CADM2* used were Forward primer (5’-CCTGCCAATCTGTGTGTGCT-3’) and Reverse primer (5’-CCATGGCCCACATGCTCTAT-3’). Enrichment was calculated as percentage of input using the 2^-ΔΔCt^ method and normalized to IgG controls.

### Immunohistochemistry

Formalin-fixed paraffin-embedded (FFPE) sections (5 μm) from ESCC tissues (adjacent normal, primary tumor, lymph node metastases) underwent deparaffinization and antigen retrieval in citrate buffer (pH 6.0) at 95 °C for 15 min. Endogenous peroxidase was blocked with 3% H_2_O_2_ for 10 min. Sections were incubated overnight at 4 °C with anti-DNMT3B primary antibody (Proteintech, Wuhan, #26971-1-AP) followed by HRP-conjugated secondary antibody for 30 min at 37 °C. Diaminobenzidine was applied for chromogenic detection with hematoxylin counterstaining. Slides were evaluated independently by two experienced pathologists blinded to the clinical data. The staining intensity was scored on a scale of 0-3 (0: negative; 1: weak; 2: moderate; 3: strong). The proportion of positive cells was scored on a scale of 1-4 (1: 1-25%; 2: 26-50%; 3: 51-75%; 4: 76-100%). The final IHC score (range 0-12) was calculated by multiplying the intensity score by the proportion score. Slides were imaged at ×200 magnification.

### Statistical analysis

All data are presented as mean ± standard deviation (SD). Statistical analyses were performed using GraphPad Prism. Normality was assessed by the Shapiro-Wilk test, and homogeneity of variance was assessed by the Brown-Forsythe test. For comparisons between two groups from independent samples, an unpaired Student’s *t*-test was applied. For comparisons between paired samples from the same individual (e.g., tumor vs. matched adjacent normal tissue), a paired Student’s *t*-test was used. For comparisons across three or more independent groups, ordinary one-way ANOVA or two-way ANOVA was used, followed by Tukey’s *post-hoc* test for multiple comparisons. For comparisons involving three or more related measurements from the same subjects (e.g., adjacent normal, primary tumor, and lymph node metastasis from the same patient), repeated-measures one-way ANOVA was applied, followed by *post-hoc* paired t-tests with Bonferroni correction for pairwise comparisons. Statistical significance thresholds were defined as *P* < 0.05, with non-significant (ns) indicating *P*≥0.05.

## Results

### *ADAMTS9-AS2* exhibits tumor-suppressive functions in ESCC cells

**Figure 1:**
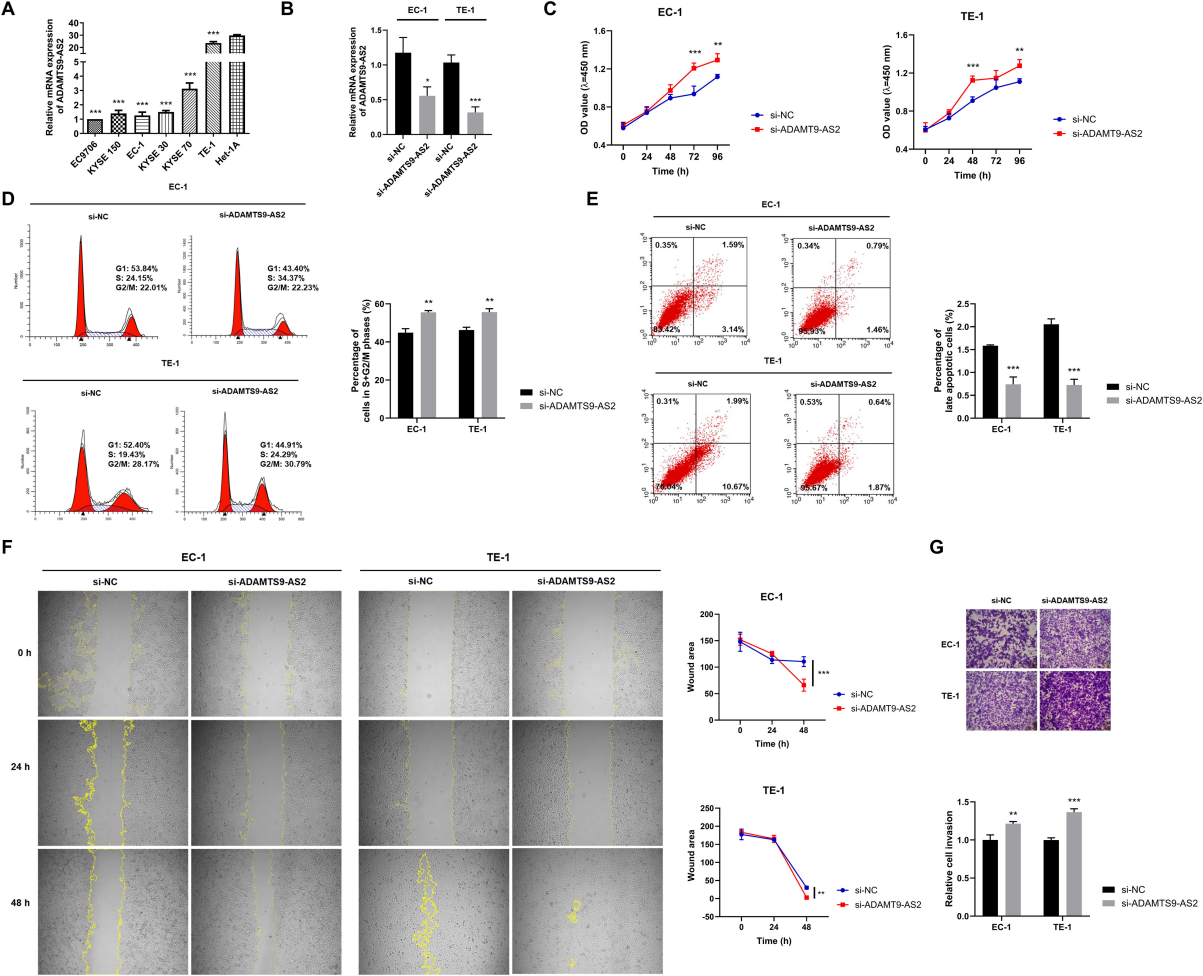
Functional characterization of ADAMTS9-AS2 in ESCC cells. **(A)** RT-qPCR analysis of *ADAMTS9-AS2* mRNA expression levels in normal human esophageal epithelial cells (Het-1A) and six ESCC cell lines (9706, KYSE150, EC-1, KYSE30, KYSE70, TE-1). Data are presented as mean ± SD (n=3 biologically independent experiments). **(B)** RT-qPCR validation of *ADAMTS9-AS2* knockdown efficiency in EC-1 and TE-1 cells transfected with siRNA targeting *ADAMTS9-AS2* (si-*ADAMTS9-AS2*) or negative control siRNA (si-NC). For EC-1, data are presented as mean ± SD (si-NC: n=8 biologically independent experiments; si-*ADAMTS9-AS2*: n=9 biologically independent experiments). For TE-1, data are presented as mean ± SD (n=9 biologically independent experiments). **(C)** CCK-8 assay assessing cell proliferation in EC-1 and TE-1 cells following *ADAMTS9-AS2* knockdown. Data are presented as mean ± SD (n=3 biologically independent experiments). **(D)** Flow cytometry analysis of cell cycle distribution in EC-1 and TE-1 cells after *ADAMTS9-AS2* knockdown. Data are presented as mean ± SD (n=3 biologically independent experiments). **(E)** Flow cytometry quantification of apoptosis rates in EC-1 and TE-1 cells transfected with si-*ADAMTS9-AS2* or si-NC. Data are presented as mean ± SD (n=3 biologically independent experiments). **(F)** Wound healing assay evaluating cell migration in EC-1 and TE-1 cells upon *ADAMTS9-AS2* knockdown. Data are presented as mean ± SD (n=3 biologically independent experiments). **(G)** Transwell invasion assay measuring invasive capacity of EC-1 and TE-1 cells after *ADAMTS9-AS2* knockdown. Data are presented as mean ± SD (n=3 biologically independent experiments). ^*^P < 0.05, ^**^P < 0.01, ^***^P < 0.001.

Given the established downregulation of *ADAMTS9-AS2* in ESCC tissues and its prognostic significance (8), we sought to delineate the functional role of *ADAMTS9-AS2* in ESCC pathogenesis. Initial screening across six ESCC cell lines (9706, KYSE150, EC-1, KYSE30, KYSE70, TE-1) and normal esophageal epithelial Het-1A cells revealed significantly reduced *ADAMTS9-AS2* mRNA expression in all cancer cell lines compared to Het-1A (**Figure 1A**; R^2^ = 0.996, *P* < 0.001; Het-1A vs. EC-1: mean diff. = 28.48, 95%*CI*: 26.34 to 30.63; Het-1A vs. TE-1: mean diff. = 6.16, 95%*CI*: 4.01 to 8.30). Based on this differential expression profile, EC-1 cells (low *ADAMTS9-AS2*) and TE-1 cells (relatively higher *ADAMTS9-AS2*) were selected for subsequent functional studies. Efficient knockdown of *ADAMTS9-AS2* was confirmed in both cell lines using specific siRNAs (si-*ADAMTS9-AS2*) compared to negative control (si-NC) (**Figure 1B**). Transfection with si-*ADAMTS9-AS2* significantly reduced *ADAMTS9-AS2* levels in EC-1 cells (mean diff.=-0.62, 95%*CI*: -1.15 to -0.09, R^2^ = 0.295, *P* = 0.024) and TE-1 cells (mean diff.=-0.72, 95%*CI*: -1.00 to -0.43, R^2^ = 0.641, *P* < 0.001).

Depletion of *ADAMTS9-AS2* significantly enhanced the proliferative capacity of both EC-1 and TE-1 cells, as evidenced by increased cell viability in CCK-8 assays (**Figure 1C**). In EC-1 cells, knockdown increased viability (R^2^ = 0.059; mean diff. = 0.114, 95%*CI*: 0.073 to 0.156), with significant effects at 72 h (mean diff.=-0.270, 95%*CI*: -0.396 to -0.144) and 96 h (mean diff.=-0.177, 95%*CI*: -0.303 to -0.051). TE-1 cells showed similar enhancement (R^2^ = 0.050; mean diff. = 0.105, 95%*CI*: 0.062 to 0.149), with significance at 48 h (mean diff.=-0.213, 95%*CI*: -0.346 to -0.081) and 96 h (mean diff.=-0.167, 95%*CI*: -0.299 to -0.034). Cell cycle analysis further demonstrated that *ADAMTS9-AS2* knockdown promoted cell cycle progression in both EC-1 (mean diff. = 10.72, 95%*CI*: 7.03 to 14.41, R^2^ = 0.942) and TE-1 cells (mean diff. = 9.41, 95%*CI*: 5.73 to 13.09, R^2^ = 0.927), indicated by a significant increase in the proportion of cells in S and G2/M phases (**Figure 1D**). Conversely, flow cytometry analysis revealed a marked reduction in apoptosis rates upon *ADAMTS9-AS2* silencing in EC-1 (mean diff.=-0.84, 95%*CI*: -1.10 to -0.58, R^2^ = 0.953) and TE-1 cells (mean diff.=-1.33, 95%*CI*: -1.60 to -1.05, R^2^ = 0.978), indicating a strong anti-apoptotic effect (**Figure 1E**). Functional assays assessing metastatic potential showed that *ADAMTS9-AS2* knockdown significantly accelerated wound closure in scratch assays in both cell lines (**Figure 1F**). In EC-1 cells, knockdown accelerated wound closure (R^2^ = 0.026; mean diff. = 9.63, 95%*CI*: -1.57 to 20.83), with significant enhancement at 48 h (mean diff. = 44.45, 95%*CI*: 19.79 to 69.10). TE-1 cells exhibited similar migratory promotion (R^2^ = 0.002; mean diff. = 6.42, 95%*CI*: -1.39 to 14.22), showing significant improvement at 48 h (mean diff. = 27.38, 95%*CI*: 10.19 to 44.56). *ADAMTS9-AS2* knockdown significantly enhanced the invasive capacity, as evidenced by increased number of invading cells in Transwell Matrigel invasion assays (**Figure 1G**). The pro-invasive effect was observed in both EC-1 cells (mean diff. = 0.215, 95%*CI*: 0.099 to 0.330, R^2^ = 0.870) and TE-1 cells (mean diff. = 0.366, 95%*CI*: 0.283 to 0.448, R^2^ = 0.974). Collectively, these findings demonstrate that *ADAMTS9-AS2* acts as a tumor suppressor in ESCC by inhibiting proliferation, promoting apoptosis, and restraining migration and invasion.

### CADM2 mediates the tumor-suppressive effects of *ADAMTS9-AS2* in ESCC

**Figure 2:**
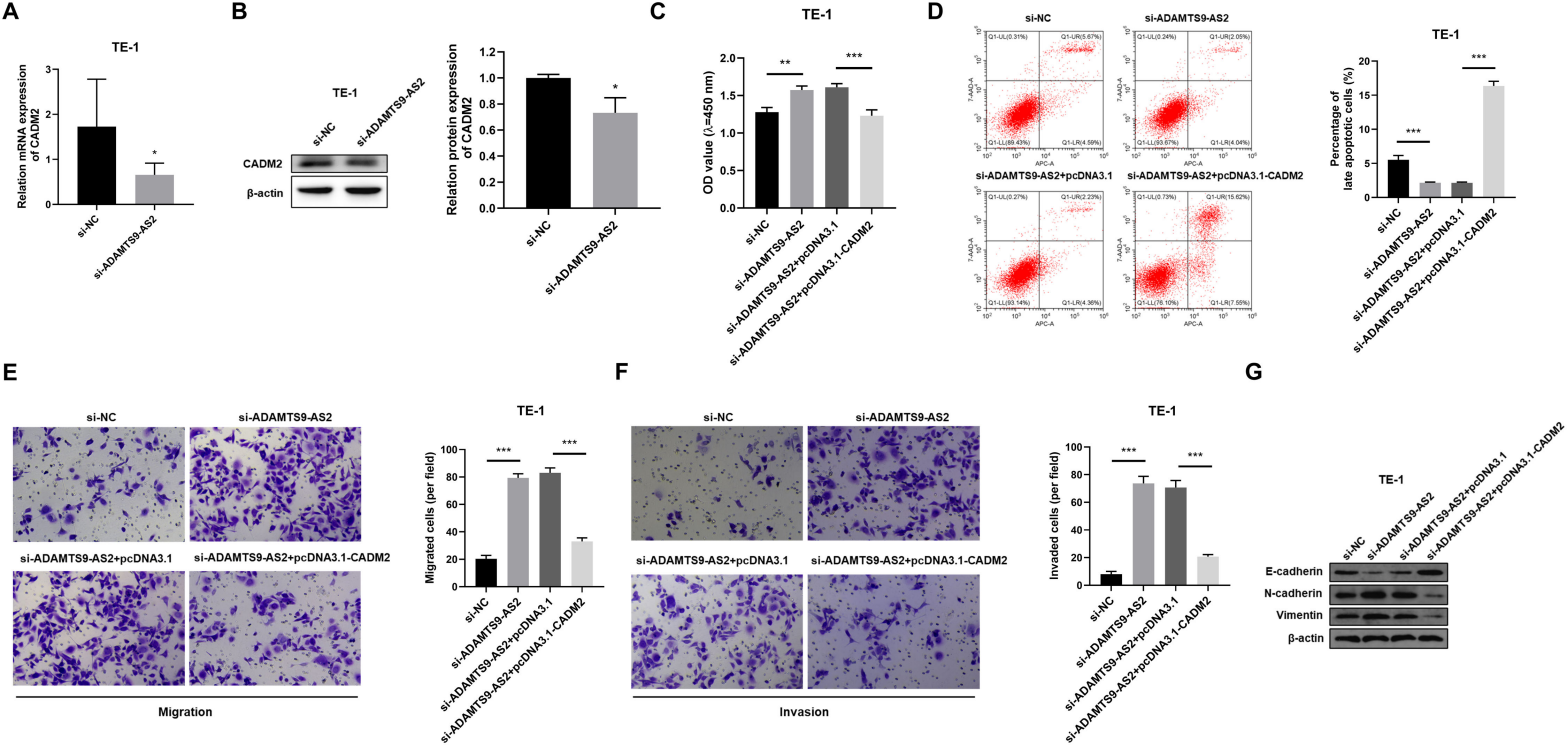
CADM2 overexpression rescues *ADAMTS9-AS2* knockdown-induced oncogenic phenotypes in TE-1 cells. **(A-B)** TE-1 cells were transfected with siRNA targeting *ADAMTS9-AS2* (si-*ADAMTS9-AS2*) or negative control siRNA (si-NC). **(A)***CADM2* mRNA expression analyzed by RT-qPCR. Data are presented as mean ± SD (si-NC: n=6 biologically independent experiments; si-*ADAMTS9-AS2*: n=8 biologically independent experiments). **(B)** CADM2 protein levels assessed by Western blot. **(C-G)** TE-1 cells were divided into four groups: si-NC, si-*ADAMTS9-AS2*, si-*ADAMTS9-AS2*+pcDNA3.1, and si-*ADAMTS9-AS2*+pcDNA3.1-*CADM2*. Data are presented as mean ± SD (n=3 biologically independent experiments). **(C)** Cell proliferation measured by CCK-8 assay. Data are presented as mean ± SD (n=3 biologically independent experiments). **(D)** Apoptosis analysis by flow cytometry using Annexin V-APC and 7-AAD dual staining. Data are presented as mean ± SD (n=3 biologically independent experiments). **(E)** Cell migration evaluated by Transwell assay (uncoated membrane). Data are presented as mean ± SD (n=3 biologically independent experiments). **(F)** Cell invasion determined by Transwell assay (Matrigel-coated membrane). Data are presented as mean ± SD (n=3 biologically independent experiments). **(G)** Protein expression of EMT markers (E-cadherin, N-cadherin, Vimentin) analyzed by Western blot. ^*^P < 0.05, ^***^P < 0.001.

To investigate whether CADM2 serves as a functional downstream effector of *ADAMTS9-AS2*, we performed rescue experiments in TE-1 cells. Knockdown of *ADAMTS9-AS2* significantly reduced both mRNA (mean diff.=-1.073, 95%*CI*: -1.905 to -0.241, R^2^ = 0.397, *P* = 0.016) and protein levels of CADM2 (mean diff.=-0.267, 95%*CI*: -0.459 to -0.076, R^2^ = 0.790, *P* = 0.018) (**Figures 2A, B**), establishing a regulatory link between these molecules.

We next examined whether CADM2 restoration could counteract *ADAMTS9-AS2* depletion-induced malignancy. One-way ANOVA revealed significant differences in proliferation among groups (R^2^ = 0.919, *P* < 0.001). While *ADAMTS9-AS2* knockdown enhanced proliferation (si-NC vs. si-*ADAMTS9-AS2*: mean diff.=-0.296, 95%*CI*: -0.458 to -0.134, *P* = 0.002), concurrent CADM2 overexpression reversed this effect, as evidenced by the significant difference between si-*ADAMTS9-AS2* and rescue groups (si-*ADAMTS9-AS2*+pcDNA3.1 vs. si-*ADAMTS9-AS2*+pcDNA3.1-CADM2: mean diff. = 0.379, 95%*CI*: 0.218 to 0.541, *P* < 0.001) (**Figure 2C**). Similarly, *ADAMTS9-AS2* silencing suppressed apoptosis (R^2^ = 0.996, *P* < 0.001), as shown by the comparison between si-NC and si-*ADAMTS9-AS2* groups (mean diff. = 3.360, 95%*CI*: 2.120 to 4.600, *P* < 0.001). Importantly, CADM2 overexpression effectively restored apoptotic rates, demonstrated by the significant difference between si-*ADAMTS9-AS2*+pcDNA3.1 and si-*ADAMTS9-AS2*+pcDNA3.1-CADM2 groups (mean diff.=-14.21, 95%*CI*: -15.45 to -12.97, *P* < 0.001) (**Figure 2D**). In addition, metastatic phenotypes were likewise modulated. *ADAMTS9-AS2* knockdown promoted migration (R^2^ = 0.992, *P* < 0.001) and invasion (R^2^ = 0.989, *P* < 0.001). Specifically, *ADAMTS9-AS2* depletion enhanced migration (si-NC vs. si-*ADAMTS9-AS2*: mean diff.=-59.00, 95%*CI*: -66.81 to -51.19) and invasion (si-NC vs. si-*ADAMTS9-AS2*: mean diff.=-65.67, 95%*CI*: -75.71 to -55.62) capabilities. Importantly, CADM2 overexpression effectively abrogated these pro-metastatic changes, as demonstrated by the significant differences between si-*ADAMTS9-AS2*+pcDNA3.1 and si-*ADAMTS9-AS2*+pcDNA3.1-CADM2 groups in both migration (mean diff. = 50.00, 95%*CI*: 42.19 to 57.81) and invasion assays (mean diff. = 50.00, 95%*CI*: 39.96 to 60.04) (**Figures 2E, F**). Mechanistically, *ADAMTS9-AS2* knockdown induced EMT, evidenced by decreased E-cadherin and increased N-cadherin/Vimentin expression. CADM2 overexpression reversed these EMT marker alterations (**Figure 2G**). These data demonstrate that CADM2 is a critical mediator through which *ADAMTS9-AS2* constrains ESCC progression by inhibiting proliferation, restoring apoptosis sensitivity, suppressing metastasis, and maintaining epithelial phenotype.

### *ADAMTS9-AS2* influences methylation patterns of *CADM2* in ESCC

**Figure 3:**
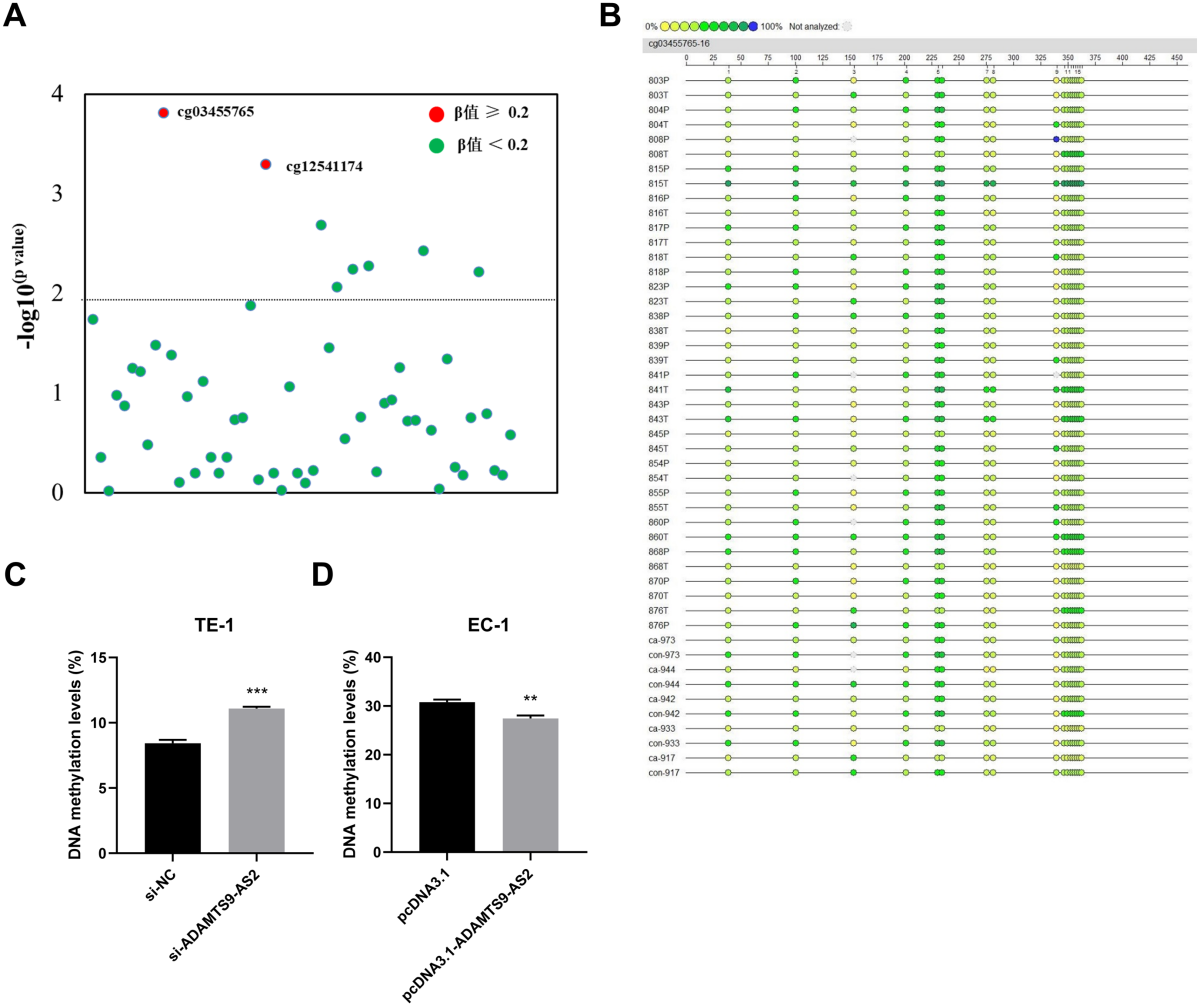
Identification of the CADM2 methylation and its regulation by *ADAMTS9-AS2*. **(A)** Genome-wide methylation profiling of 5 paired ESCC tumor (T) and adjacent normal (N) tissues displays β-values of 54 CpG sites within the *CADM2* gene locus. **(B)** Validation of cg03455765 methylation in 24 paired ESCC tumor (T) samples and para-cancerous (P) tissues by MassARRAY EpiTYPER analysis (Agena Bioscience). Heatmap visualization shows methylation levels (0%-100%, color-coded) at 17 CpG sites (horizontal axis) within the amplified region of cg03455765. Grey indicates non-analyzed sites. **(C)** Pyrosequencing analysis of *CADM2* methylation in TE-1 cells transfected with si-*ADAMTS9-AS2* or si-NC. Data are presented as mean ± SD (n=3 biologically independent experiments). **(D)***CADM2* methylation levels in TE-1 cells transfected with *ADAMTS9-AS2* overexpression vector or empty vector. Data are presented as mean ± SD (n=3 biologically independent experiments). ^**^P < 0.01, ^***^P < 0.001.

To investigate epigenetic dysregulation of CADM2 in ESCC, we performed genome-wide methylation screening in 5 paired ESCC tumor and adjacent normal tissues. Quality control analyses confirmed data reliability, showing clear sample separation by PCA (**Supplementary Figure 1**) and high within-group consistency in both methylation (**Supplementary Figure 2**) and transcriptomic data (**Supplementary Figure 3**). A total of 54 CpG sites within the *CADM2* locus were identified (**Figure 3A**). Among these, the site cg03455765 was selected for further investigation as it exhibited the most substantial hypomethylation (Δβ=-0.296, adjusted *P* = 0.031) in tumors, meeting our predefined thresholds of |Δβ|≥0.2 and adjusted *P* < 0.05 (**Supplementary Table 3**). This finding was validated in an expanded cohort of 24 ESCC patients using MassARRAY technology. The analysis covered 17 CpG sites around cg03455765, and consistent hypomethylation at cg03455765 was observed, particularly at CpG_2 (*P* = 0.002) and CpG_4 (*P* = 0.002) sites (**Table 2**, **Figure 3B**). This observation is particularly noteworthy in the context of gene body methylation, which is generally associated with active transcription. The hypomethylation we identified at the CADM2 gene body is therefore consistent with a model whereby loss of methylation in gene body region contributes to transcriptional suppression, a concept strongly supported by prior research (12).

**Table 2:** Site-specific methylation levels of 17 CpG sites within the cg03455765 region in ESCC tumors versus adjacent normal tissues.

CpG	Average methylation level (Case)	Average methylation level (Control)	*P* value
cg03455765 CpG_1	0.336	0.363	0.453
cg03455765 CpG_2	0.324	0.419	0.002
cg03455765 CpG_3	0.267	0.241	0.565
cg03455765 CpG_4	0.325	0.418	0.002
cg03455765 CpG_5.6	0.502	0.547	0.203
cg03455765 CpG_7.8	0.251	0.269	0.575
cg03455765 CpG_9	0.232	0.163	0.409
cg03455765 CpG_10-17	0.366	0.323	0.234

We next examined whether *ADAMTS9-AS2* modulates *CADM2* methylation. Pyrosequencing of CpG-rich region within *CADM2* revealed that *ADAMTS9-AS2* knockdown significantly increased *CADM2* CpG methylation compared to controls (mean diff. = 2.667, 95%*CI*: 2.187 to 3.146, R^2^ = 0.984, *P* < 0.001) (**Figure 3C**). Conversely, *ADAMTS9-AS2* overexpression reduced methylation levels (mean diff.=-3.327, 95%*CI*: -4.631 to -2.023, R^2^ = 0.926, *P* = 0.002) (**Figure 3D**). These results demonstrate that *ADAMTS9-AS2* influences CpG methylation patterns of *CADM2*.

### *ADAMTS9-AS2* constrains DNMT3B to prevent *CADM2* epigenetic silencing

**Figure 4:**
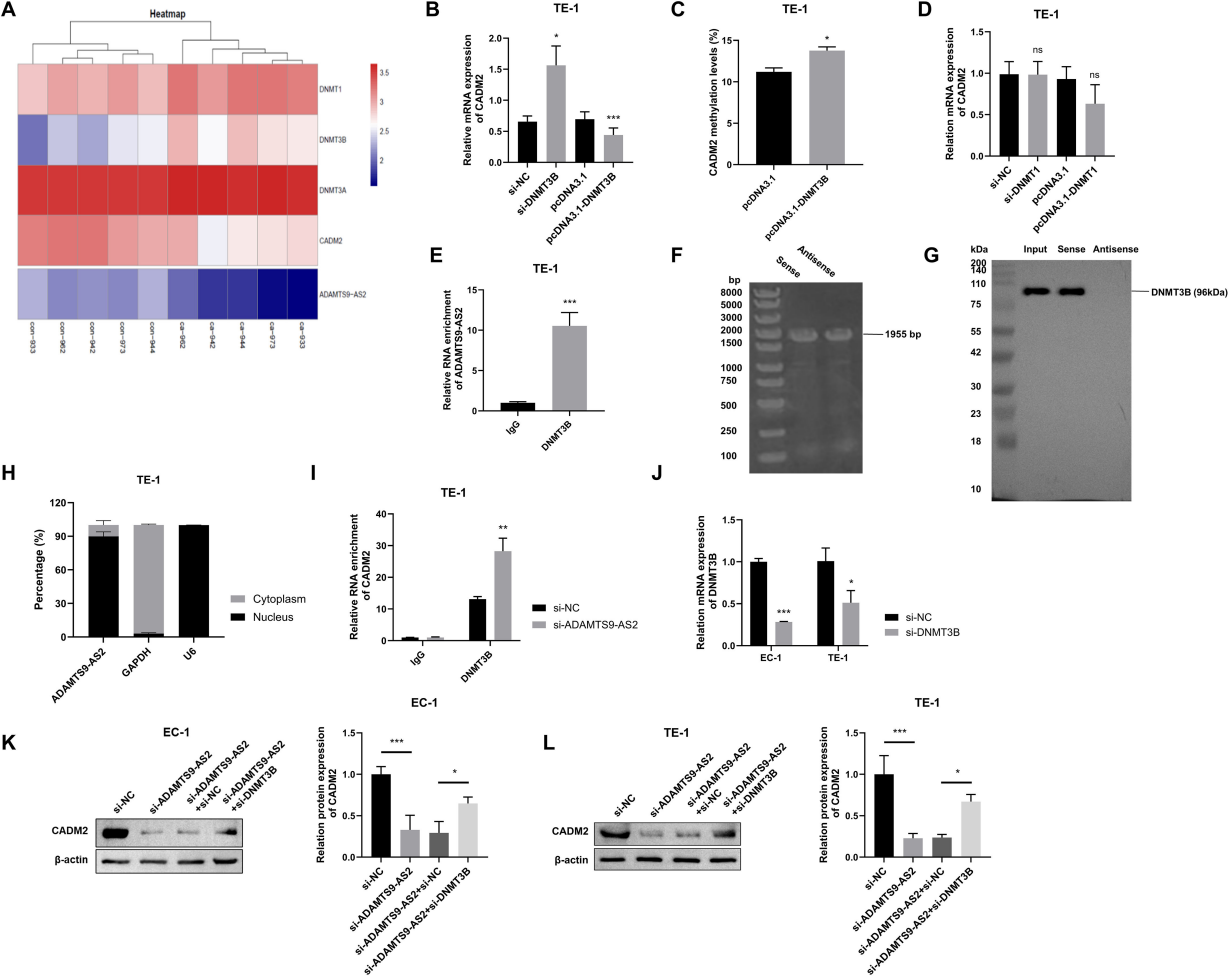
*ADAMTS9-AS2* constrains DNMT3B to prevent *CADM2* epigenetic silencing. **(A)** mRNA microarray heatmap of DNMT family, *CADM2*, and *ADAMTS9-AS2* expression in 5 paired ESCC tumors and adjacent normal tissues. Tumor samples are labeled ca- (e.g., ca-933), adjacent normal tissues labeled con- (e.g., con-933). Blue indicates low expression, and red indicates high expression. Data are derived from n=5 biologically independent patient samples per group. **(B)** RT-qPCR analysis of *CADM2* mRNA in TE-1 cells after DNMT3B knockdown or overexpression. Data are presented as mean ± SD (si-NC: n=8; si-DNMT3B: n=6; pcDNA3.1: n=6; pcDNA3.1-DNMT3B: n=6 biologically independent experiments). **(C)***CADM2* methylation levels in TE-1 cells transfected with empty vector (pcDNA3.1) or DNMT3B overexpression plasmid (pcDNA3.1-*DNMT3B*). Data are presented as mean ± SD (n=3 biologically independent experiments). **(D)***CADM2* mRNA levels in TE-1 cells following DNMT1 modulation. Data are presented as mean ± SD (si-NC: n=8; si-DNMT1: n=6; pcDNA3.1: n=8; pcDNA3.1-DNMT1: n=4 biologically independent experiments). **(E)** RIP assay detecting DNMT3B-*ADAMTS9-AS2* binding. Data are presented as mean ± SD (n=3 biologically independent experiments). **(F)** Validation of *in vitro* transcribed biotinylated RNA probes by streptavidin-HRP blot. Sense and antisense *ADAMTS9-AS2* RNA probes show efficient biotinylation. **(G)** RNA pull-down assay demonstrating direct binding between *ADAMTS9-AS2* and DNMT3B. Western blot using anti-DNMT3B antibody shows specific enrichment of DNMT3B protein pulled down by the sense *ADAMTS9-AS2* probe, but not by the antisense control probe. **(H)** Subcellular localization of *ADAMTS9-AS2* in TE-1 cells. Nuclear and cytoplasmic RNA fractions were isolated and analyzed by RT-qPCR. *ADAMTS9-AS2* is predominantly localized in the nucleus, with detectable expression in the cytoplasm. U6 snRNA and GAPDH mRNA served as nuclear and cytoplasmic controls, respectively. Data are presented as mean ± SD (n=3 biologically independent experiments). **(I)** ChIP-qPCR measuring DNMT3B enrichment at the *CADM2* gene post-*ADAMTS9-AS2* knockdown. Data are presented as mean ± SD (n=3 biologically independent experiments). **(J)** DNMT3B knockdown efficiency validation by RT-qPCR. Data are presented as mean ± SD (n=3 biologically independent experiments). **(K, L)** EC-1 and TE-1 cells were divided into four groups: si-NC, si-*ADAMTS9-AS2*, si-*ADAMTS9-AS2*+sh-NC, and si-*ADAMTS9-AS2*+sh-*DNMT3B*. Western blot analysis of CADM2 protein rescue after co-transfection with si-*ADAMTS9-AS2* and sh-*DNMT3B*. Data are presented as mean ± SD (n=3 biologically independent experiments). ns: not significant, ^*^P < 0.05, ^**^P < 0.01, ^***^P < 0.001.

Transcriptomic profiling via mRNA microarray demonstrated tumor-specific upregulation of epigenetic regulators. *DNMT3B* (FC = 3.49, *P* = 0.0047) and *DNMT1* (FC = 2.58, *P* = 0.0073) exhibited significantly higher expression in tumors versus adjacent normal tissues (**Figure 4A**, **Table 3**), while *CADM2* (FC = 4.33, *P* = 0.0055) and *ADAMTS9-AS2* (FC = 2.31, *P* = 0.0117) showed tumor-specific suppression, implying their reciprocal relationship with DNMTs. Conversely, *DNMT3A* displayed comparable expression with no consistent differential pattern across tissues (**Figure 4A**). Functional studies demonstrated that DNMT3B bidirectionally regulates *CADM2* expression. Knockdown of DNMT3B increased *CADM2* mRNA levels (mean diff. = 0.906, 95%*CI*: 0.280 to 1.532, R^2^ = 0.453, *P* = 0.018), whereas its overexpression suppressed *CADM2* (mean diff.=-0.256, 95%*CI*: -0.622 to -0.110, R^2^ = 0.196, *P* < 0.001) (**Figure 4B**). Consistently, overexpression of DNMT3B in TE-1 cells led to a significant increase in the methylation levels of the *CADM2* compared to the empty vector control (mean diff.=-2.557%, 95%*CI*: -4.746 to -0.367, R^2^ = 0.927, *P* = 0.037) (**Figure 4C**). Notably, DNMT1 modulation did not alter *CADM2* expression (siRNA knockdown: mean diff.=-0.005, 95%*CI*: -0.493 to 0.483, R^2^<0.001, *P* = 0.982; overexpression: mean diff.=-0.298, 95%*CI*: -0.890 to 0.294, R^2^ = 0.112, *P* = 0.288) (**Figure 4D**). Based on these transcriptomic correlations and functional validation, DNMT3B was prioritized for mechanistic investigation of *CADM2* epigenetic regulation.

**Table 3:** Differentially expressed genes related to the *ADAMTS9-AS2*/DNMT3B/CADM2 axis in ESCC tumors versus adjacent normal tissues.

Probe Name	Gene Symbol	Regulation	*P* value	FC (abs)
A_23_P154500	DNMT3A	up	0.063543	1.159372524
A_23_P28953	DNMT3B	up	0.00471	3.485189181
A_33_P3329187	DNMT1	up	0.007313	2.577836435
A_33_P3353210	CADM2	down	0.005549	4.330162891
p11845	*ADAMTS9-AS2*	down	0.011706	2.311065591

RIP assay confirmed the binding relation between DNMT3B protein and *ADAMTS9-AS2* RNA (mean diff. = 9.542, 95%*CI*: 6.885 to 12.200, R^2^ = 0.961, *P* < 0.001) (**Figure 4E**). To further substantiate a direct interaction, we performed RNA pull-down assays using *in vitro* transcribed, biotinylated *ADAMTS9-AS2* RNA. Successful probe synthesis was confirmed by gel electrophoresis (**Figure 4F**). Subsequent Western blot analysis demonstrated that DNMT3B protein was specifically enriched by the sense *ADAMTS9-AS2* probe, but not by the antisense control probe, confirming a direct RNA-protein interaction (**Figure 4G**). To investigate the subcellular context of this interaction, we performed cellular fractionation. RT-qPCR analysis revealed that *ADAMTS9-AS2* was predominantly localized in the nucleus (mean nucleus% vs. cytoplasm% diff.=-79.85, 95%*CI*: -88.87 to -70.82, R^2^ = 0.993), with detectable levels present in the cytoplasm (**Figure 4H**). This distribution supports a model where *ADAMTS9-AS2* can sequester DNMT3B in both compartments, potentially preventing its nuclear translocation or chromatin engagement. Consistent with this, ChIP assay showed that *ADAMTS9-AS2* knockdown enhanced DNMT3B occupancy at the *CADM2* gene (mean diff. = 15.13, 95%*CI*: 8.420 to 21.84, R^2^ = 0.907, *P* = 0.003) (**Figure 4I**), indicating *ADAMTS9-AS2* constrains DNMT3B-mediated epigenetic silencing. Rescue experiments validated this axis. Efficient DNMT3B knockdown (EC-1: mean diff.=-0.718, 95%*CI*: -0.781 to -0.654, R^2^ = 0.996; TE-1: mean diff.=-0.497, 95%*CI*: -0.838 to -0.155, R^2^ = 0.803) (**Figure 4J**) reversed *ADAMTS9-AS2* silencing-induced CADM2 suppression in both EC-1 and TE-1 cells, as demonstrated by the significant upregulation of CADM2 upon concurrent DNMT3B knockdown (EC-1: si-*ADAMTS9-AS2*+si-NC vs. si-*ADAMTS9-AS2*+si-DNMT3B: mean diff.=-0.356, 95%*CI*: -0.688 to -0.024, R^2^ = 0.883; TE-1: mean diff.=-0.434, 95%*CI*: -0.762 to -0.106, R^2^ = 0.909) (**Figures 4K, L**). These results establish that *ADAMTS9-AS2* sequesters DNMT3B to influence *CADM2* methylation, revealing a key tumor-suppressive mechanism in ESCC.

### DNMT3B protein expression in ESCC tissues by IHC

**Figure 5:**
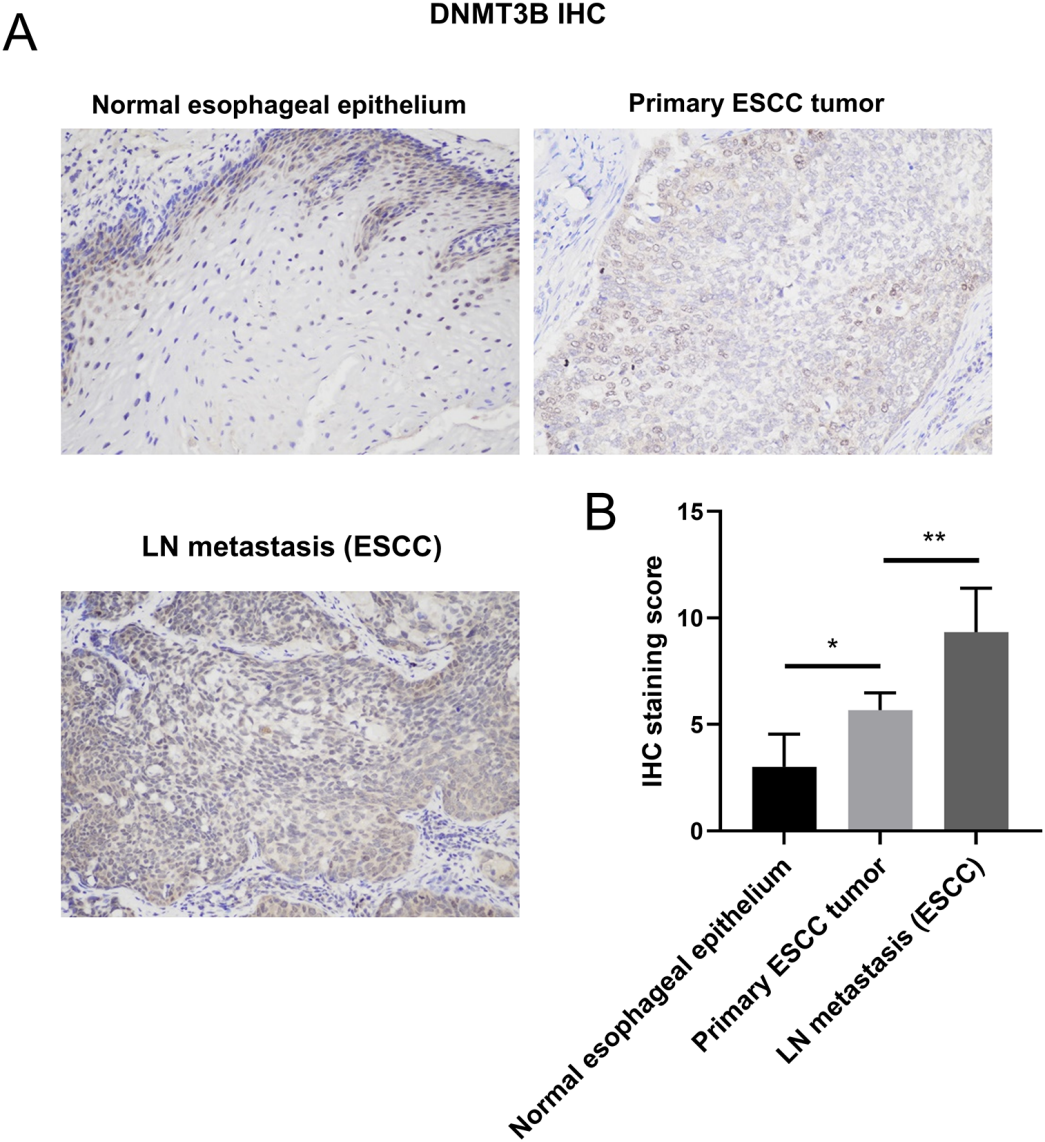
DNMT3B protein expression in ESCC tissues by IHC. **(A)** Representative IHC images of DNMT3B staining in adjacent normal esophageal epithelium, primary ESCC tumor tissues, and lymph node-positive metastatic lesions from ESCC patients. Magnification: ×200. **(B)** Quantitative analysis of DNMT3B IHC staining score in the three tissue groups. Data are presented as mean ± SD (n=6 biologically independent samples per group). ^*^P < 0.05, ^**^P < 0.01.

IHC analysis revealed progressive DNMT3B upregulation across ESCC disease progression (R^2^ = 0.768, *P* < 0.001). Compared to histologically normal adjacent tissues, primary ESCC tumors exhibited significantly increased DNMT3B staining (mean diff.=-2.667, 95%*CI*: -5.011 to -0.322). Strikingly, lymph node metastases demonstrated further enhanced DNMT3B expression versus primary tumors (mean diff.=-3.667, 95%*CI*: -6.011 to -1.322), with near-universal nuclear positivity in malignant cells (**Figures 5A, B**). This stepwise elevation establishes DNMT3B as a metastasis-associated epigenetic regulator in ESCC, aligning with transcriptomic data showing tumor-specific DNMT3B overexpression.

### The *ADAMTS9-AS2*/CADM2 axis is associated with immune cell infiltration in ESCC

**Figure 6:**
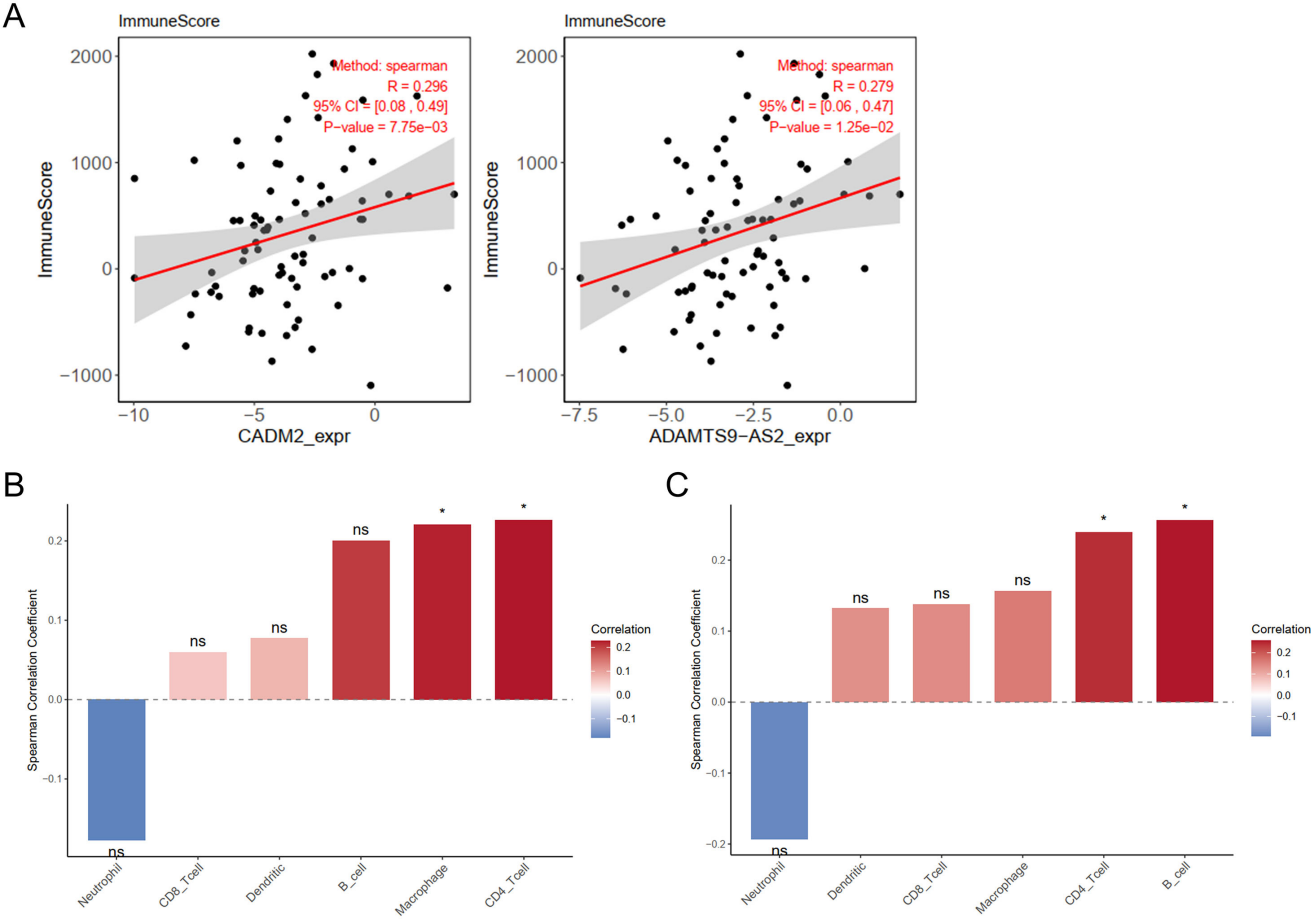
Correlation of the *ADAMTS9-AS2*/CADM2 axis with immune infiltration in ESCC. **(A)** Scatter plots showing the correlation between gene expression and the ESTIMATE ImmuneScore in ESCC samples from a public cohort. Left panel: CADM2 mRNA expression (R = 0.296, *P* = 7.75e-3). Right panel: *ADAMTS9-AS2* expression (R = 0.279, *P* = 1.25e-2). The solid line indicates the linear regression fit, and the shaded area represents the 95% confidence interval. **(B)** Spearman correlation analysis between CADM2 expression and the estimated abundance of six major immune cell types within the tumor microenvironment, as calculated by the TIMER algorithm. Data are presented as correlation coefficients (R) with corresponding *P*-values indicated. **(C)** Spearman correlation analysis between *ADAMTS9-AS2* expression and immune cell infiltration levels. Data are presented as correlation coefficients (R) with corresponding *P*-values indicated. ns: not significant, ^*^P < 0.05.

To assess the potential immunomodulatory role of the identified axis, we performed immune correlation analyses using transcriptomic data from a public ESCC cohort. The ESTIMATE algorithm was employed to evaluate overall immune infiltration. We found that expression levels of both CADM2 (R = 0.296, *P* = 7.75e-3) and *ADAMTS9-AS2* (R = 0.279, *P* = 1.25e-2) were significantly and positively correlated with the ESTIMATE ImmuneScore (**Figure 6A**), indicating an association with a more immune-infiltrated tumor microenvironment. To characterize the specific immune cell types involved, we performed immune deconvolution using the TIMER algorithm. CADM2 expression showed a significant positive correlation with the infiltration level of CD4^+^ T cells (**Figure 6B**). A similar positive correlation trend between *ADAMTS9-AS2* expression and CD4^+^ T cell abundance was also observed (**Figure 6C**). These bioinformatics analyses suggest that the activity of the *ADAMTS9-AS2*/CADM2 axis is linked to the presence of specific adaptive immune cells within the ESCC tumor microenvironment.

## Discussion

ESCC remains a global health burden with dismal survival rates in advanced stages due to metastatic recurrence. Epigenetic dysregulation plays a pivotal role in this process particularly through DNA methyltransferase-mediated silencing of tumor suppressors (13, 14). The DNMT family consists of three key members DNMT1 maintains methylation patterns during replication, while DNMT3A and DNMT3B establish *de novo* methylation (15). Although DNMT1 and DNMT3A functions in ESCC have been partially characterized (4, 5), DNMT3B’s role remained enigmatic despite its frequent dysregulation in this malignancy. Emerging evidence now reveals DNMT3B actively silences tumor-suppressive miRNAs to drive ESCC pathogenesis. Xiao et al. reported that DNMT3B suppresses *miR-493-5p* to promote VEGF-mediated angiogenesis (16). Yang et al. demonstrated that DNMT3B methylates *miR-149* promoter to activate RNF2/Wnt/β-catenin signaling (17). However, lncRNA-mediated regulation of DNMT3B activity, particularly its sequestration to prevent tumor suppressor methylation, remained unexplored prior to this study.

LncRNAs represent crucial regulators in cancer pathogenesis (18, 19). Most studies focus on their roles in transcriptional regulation or miRNA sponging (20, 21). For instance, *DANCR* promotes ESCC progression by sponging *miR-3193* to regulate DDIT3 expression (22), while *TPT1-AS1* drives ESCC metastasis through the *miR-26a*/HMGA1 axis (23). In contrast, direct modulation of DNMT activity by lncRNAs represents an emerging area with limited exploration. Qi et al. demonstrated that lncRNA *DBCCR1–003* binds DNMT1 and prevents DNMT1-mediated methylation of the tumor suppressor *DBCCR1* in bladder cancer (24). *ADAMTS9-AS2* is a multifunctional lncRNA with context-dependent roles in cancer (11). In ESCC, *ADAMTS9-AS2* exerts its tumor-suppressive role by acting as a molecular sponge for miR-196b-5p, thereby inhibiting the malignant progression of cancer cells (25). Importantly, our previous clinical study established that both *ADAMTS9-AS2* and CADM2 serve as independent prognostic indicators in ESCC, with their expression levels significantly correlated with patient survival outcomes (8). Although isolated studies suggest potential epigenetic functions, such as mediating *CDH3* methylation in ESCC (26) or being regulated by DNMT1 in glioma (27). The direct evidence of *ADAMTS9-AS2* modulating DNMT activity remains absent. This knowledge gap motivated our investigation into its interaction with DNMT3B.

CADM2 functions as a critical tumor suppressor in ESCC (8). Prior research demonstrated its suppression through miRNA pathways including *miR-21-5p*-mediated proliferation promotion (9) and *miR-182-5p*-driven invasion acceleration (10). Moreover, CADM2 is significantly downregulated in ESCC tissues and correlates with poor prognosis, with its expression showing positive synchrony with *ADAMTS9-AS2* as demonstrated by Shen et al. (8) However, the potential regulation of CADM2 through DNA methylation remained unexplored.

This study bridges these knowledge gaps by identifying a novel *ADAMTS9-AS2*/DNMT3B/*CADM2* regulatory axis. We demonstrate that *ADAMTS9-AS2* acts as a decoy by physically binding and sequestering DNMT3B, thereby preventing its access to the *CADM2* gene. Our findings establish DNMT3B as the specific DNMT isoform responsible for CADM2 silencing in ESCC. Therapeutically this axis offers two targeted strategies restoring *ADAMTS9-AS2* expression or inhibiting DNMT3B activity both potentially capable of reactivating *CADM2* while avoiding systemic toxicity associated with pan-DNMT inhibitors.

Our bioinformatic analyses further suggest an immunomodulatory role for this epigenetic axis. Expression of both *ADAMTS9-AS2* and CADM2 positively correlates with immune infiltration, particularly CD4^+^ T cell abundance, in the ESCC microenvironment. This implies that silencing of this axis may contribute to an immunosuppressive niche, potentially through impaired immune cell interactions mediated by the adhesion molecule CADM2. A promising future direction is to investigate whether restoring this axis can synergize with immune checkpoint blockade, offering a combined epigenetic-immunotherapeutic strategy.

These findings underscore the translational potential of the ADAMTS9-AS2/DNMT3B/CADM2 axis. For future ESCC patients, assessing ADAMTS9-AS2 expression or CADM2 methylation status could provide a prognostic biomarker to identify high-risk individuals for metastasis, enabling more personalized surveillance strategies. Therapeutically, the development of ADAMTS9-AS2 mimetics or specific DNMT3B inhibitors represents a promising avenue for novel epigenetic therapy aimed at suppressing metastasis. To advance these findings towards clinical application, future work must focus on validating this regulatory axis in larger prospective cohorts, developing and testing targeted DNMT3B inhibitors in preclinical models, and exploring their potential synergy with existing treatments such as immunotherapy.

Study limitations warrant acknowledgment. First, the initial genome-wide screening cohort was limited in size (n=5 pairs), which is typical for discovery-phase, high-cost omics studies. However, key findings were rigorously validated in a larger, independent cohort. Second, while DNMT3B knockdown restored CADM2 expression, phenotypic rescue experiments remain incomplete without metastasis reversal data. Third, the structural basis of *ADAMTS9-AS2*-DNMT3B binding requires further characterization to inform therapeutic development. Finally, *in vivo* validation using metastatic models would strengthen clinical relevance.

This work establishes *ADAMTS9-AS2* as a metastasis suppressor that functions as an epigenetic brake by sequestering DNMT3B and preventing its methylation of the *CADM2*. The *ADAMTS9-AS2*/DNMT3B/*CADM2* axis represents a previously unrecognized target in ESCC metastasis offering novel therapeutic opportunities against this lethal malignancy.

## Data Availability

The original contributions presented in the study are publicly available. This data can be found here: Gene Expression Omnibus (GEO), accession number GSE322956.

## Supplementary material

Supplementary Table 3 Differentially methylated positions within the CADM2 locus identified by genome-wide screening.

Supplementary Table 4 Sample metadata and raw data file inventory for genome-wide methylation analysis.

Supplementary Figure 1 PCA of genome-wide DNA methylation profiles.

Supplementary Figure 2 Inter-sample correlation heatmap of DNA methylation data.

Supplementary Figure 3 Sample correlation heatmap of gene expression data.
