# Supplementary figures and images for: ADAMTS9-AS2 acts as an epigenetic brake to constrain DNMT3B-mediated CADM2 silencing in esophageal squamous cell carcinoma metastasis
Source: Front Immunol. 2026 Mar 6;17:1752827. doi: 10.3389/fimmu.2026.1752827 (PMC13003458; doi:10.3389/fimmu.2026.1752827)

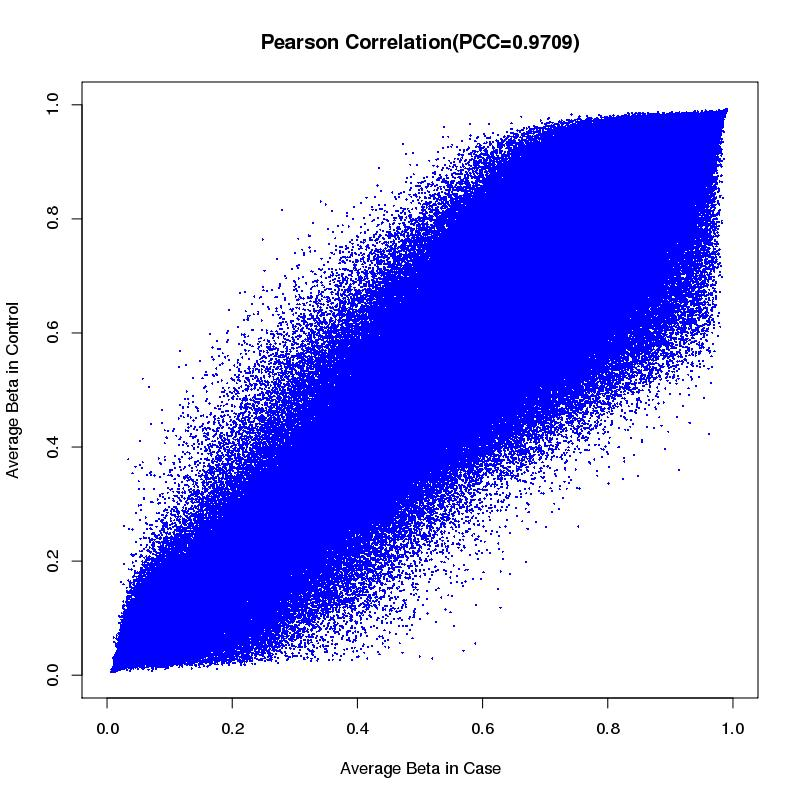

Supplement: Supplementary Figure 1 — PCA of genome-wide DNA methylation profiles. [file Image1.tif]

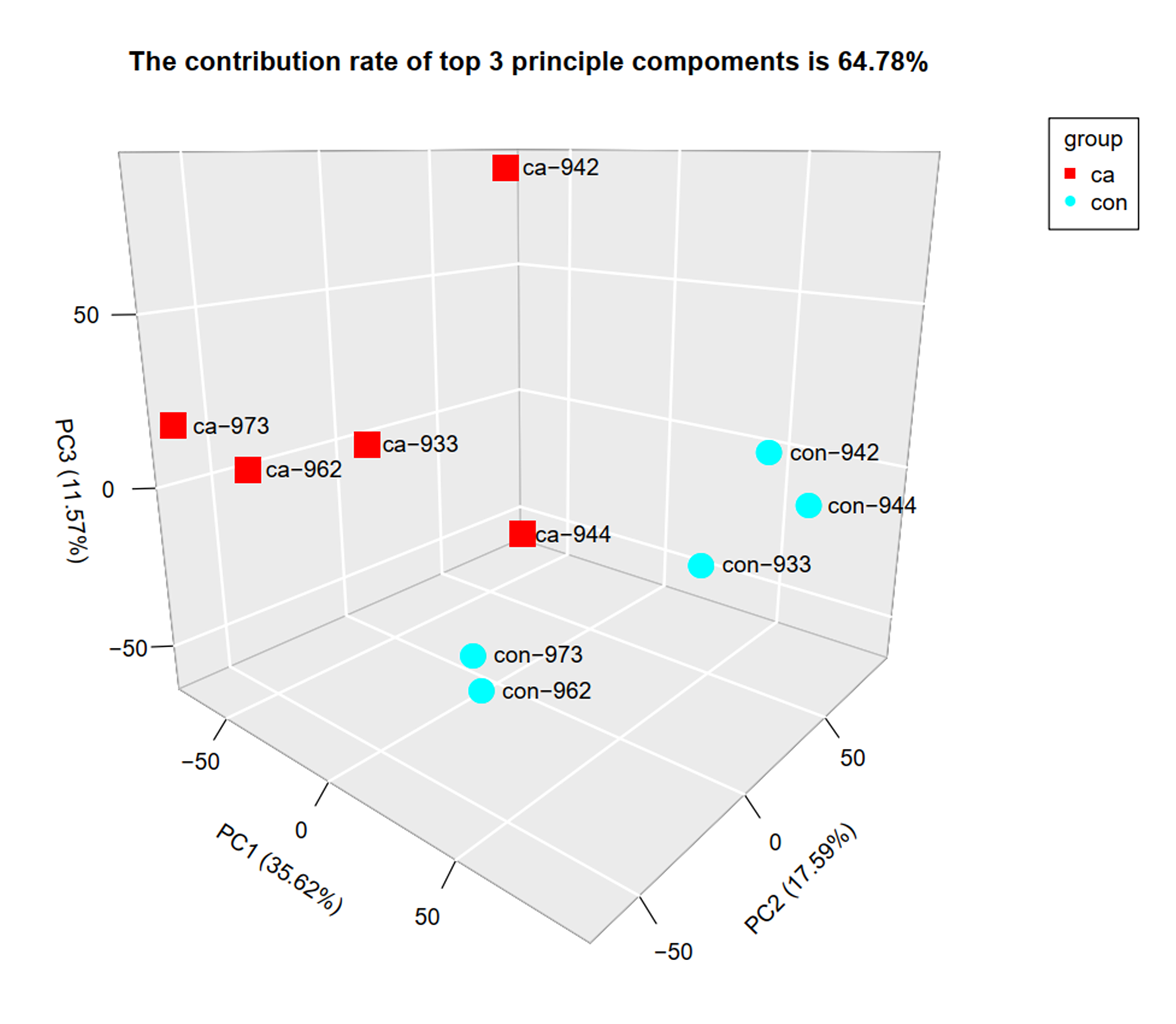

Supplement: Supplementary Figure 2 — Inter-sample correlation heatmap of DNA methylation data. [file Image2.tif]

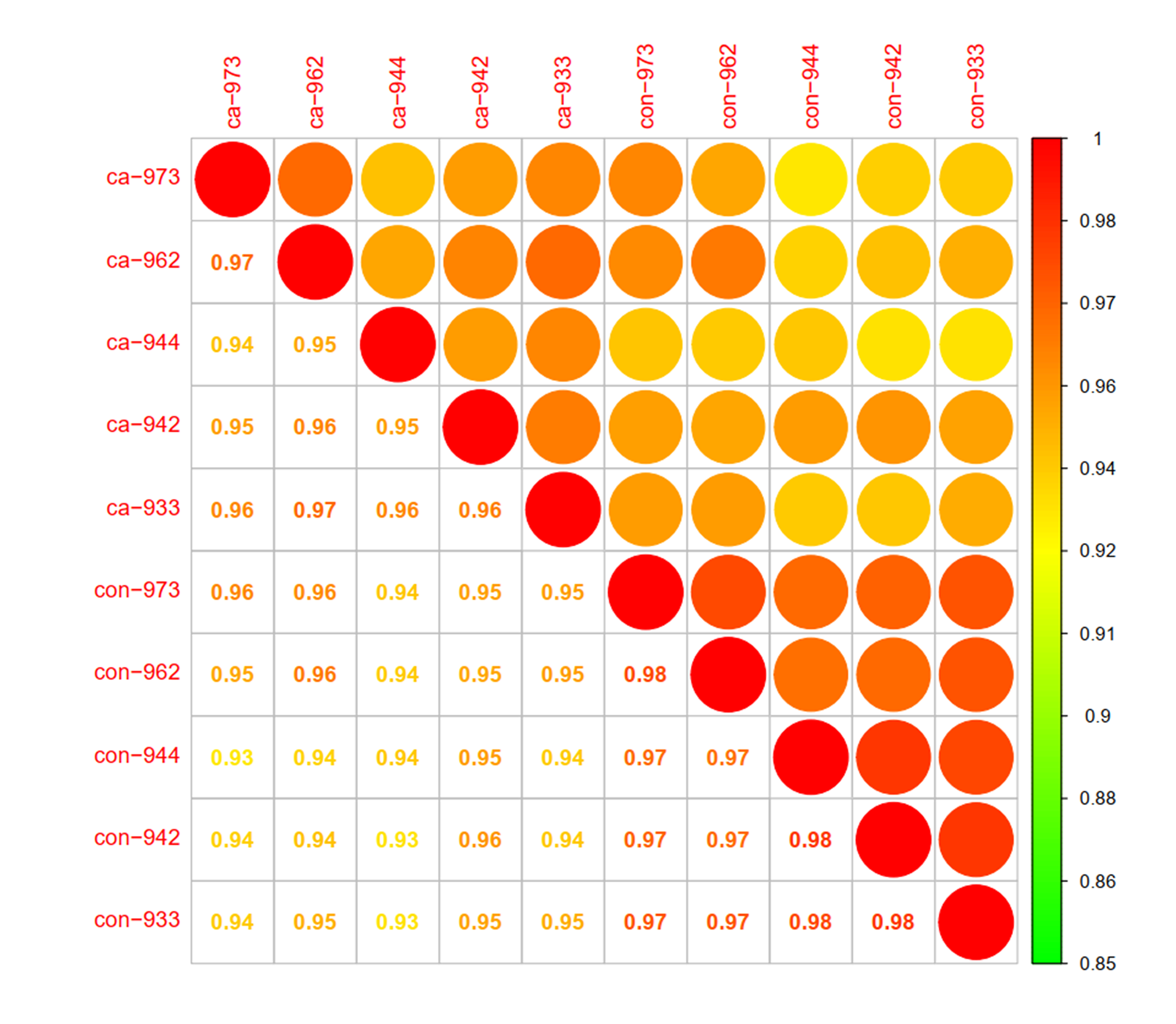

Supplement: Supplementary Figure 3 — Sample correlation heatmap of gene expression data. [file Image3.tif]
